# Association between children’s caregivers time preferences and childhood overweight and obesity in Mexico

**DOI:** 10.1371/journal.pone.0283455

**Published:** 2024-03-07

**Authors:** Mariana Molina, Godefroy Emmanuel Guindon, Laura N. Anderson, Jean-Eric Tarride

**Affiliations:** 1 Department of Health Research Methods, Evidence, and Impact, McMaster University, Hamilton, Ontario, Canada; 2 Centre for Health Economics and Policy Analysis, McMaster University, Hamilton, Ontario, Canada; 3 Department of Economics, McMaster University, Hamilton, Ontario, Canada; 4 The Hospital for Sick Children Research Institute, Child Health Evaluative Sciences, Toronto, Ontario, Canada; 5 The Research Institute of St. Joe’s Hamilton, St. Joseph’s Healthcare Hamilton, Hamilton, ON, Canada; 6 McMaster Chair in Health Technology Management Hamilton, Hamilton, ON, Canada; Yale University, UNITED STATES

## Abstract

**Background:**

Parents or children’s primary caregivers are a key influence on child weight as both decision makers and role models for eating patterns, physical activity, and other social behaviors. It is unknown whether caregivers’ time preferences are associated with overweight or obesity in children. The primary objective was to estimate the association between parents’ or caregivers’ time preferences and children having overweight or obesity in Mexico.

**Methods:**

A cross-sectional study was conducted using a representative survey of the Mexican population. A multinomial logistic model was used to examine the association between parents’ or caregivers’ time preferences (patience and time consistency) and child overweight or obesity, adjusting for potential confounders.

**Results:**

The study included 9,102 children (mean age 10, 43% female) and 5,842 caregivers (mean age 37; 95% female). Intertemporal preference was strongly associated with increased odds of overweight or obesity in children. A medium patient caregiver had higher odds of having overweight (adjusted OR: 1.73; 95% CI: 1.19, 2.52). Similarly, having a caregiver with a present (OR: 2.52; 95% CI: 1.72, 3.70) or future bias (OR: 1.48; 95% CI: 1.11, 1.98) was associated with higher odds of obesity.

**Conclusion:**

Caregivers’ time preferences were associated with having overweight and obesity in children and should be considered when developing policies to reduce children’s obesity status.

## 1. Introduction

Due to its high prevalence, associated morbidity, and associated costs, obesity is a major public health issue. It is well understood that obesity is a multifactorial problem, combining biological, environmental, social, behavioral, and economic elements [1, 2] as well as lifestyle decisions [3–5]. While policy decisions and environmental factors influence healthy or unhealthy choices, individuals’ time preferences also shape decisions [3]. The term ‘time preferences’ (also sometimes referred to as time discounting) describes ‘the extent to which individuals value benefits and costs that arise in the future differently (usually less) than if they arise today’ [6]. In related terms, individuals discount future rewards as they are perceived as less valuable than the same reward now [7, 8]. Individuals with a higher discount rate tend to prefer short-term gratifications, while individuals with a low discount rate favor long-term and possibly larger rewards [7, 9, 10]. These time preferences (e.g., an inclination for the present or the future) have been recognized to play an important role in understanding individuals’ health habits or eating behavior and obesity risk [10–13]. Consuming highly palatable unhealthy food provides immediate pleasure but has negative consequences in the long term, whereas healthy eating or exercise has distant benefits but an immediate cost. For example, people could value more the immediate pleasure from eating over future healthy aging. This could explain why some individuals would prefer smaller immediate rewards over larger rewards available after a delay [14].

The results of a recent systematic review of 41 studies exploring the relationship between unhealthy diets, obesity, and time discounting found that high-time discount rates (i.e., individuals valuing the present more than the future) were significant risk factors for unhealthy diets [3]. However, most studies assessed in this systematic review focussed primarily on adults, and very few explored the impact of parents’ time preferences on childhood obesity. This is important because parents or caregivers are a key influence on child weight as decision-makers and role models for eating patterns, physical activity, and other social behaviors [15, 16]. The parental transmission of obesity has been explored as a direct effect through genetics and household environment, but also as an indirect influence given by the spread of unhealthy behaviors from parents to children [17–19]. Recently one study exploring the intergenerational effects of parents’ time preferences and children’s obesity status reported that parents with a preference for the present instead of the future had a higher likelihood of having children with obesity after adjusting for several covariates [17]. However, this study was conducted in the U.S., and the generalizability of results to other settings or countries is limited. For example, in Mexico, the prevalence of obesity in children is among the highest in the world [20], and the impact of time preferences is unknown. To fill a gap in the literature and to inform future health policies, our primary objective was to determine the association between parents’ or caregivers’ time preferences and overweight and obesity in their children in Mexico. Secondary objectives were to determine the association between caregiver’s time preferences and children’s overweight or obesity by child age and sex and to identify other factors independently associated with children’s obesity status.

## 2. Methods

### 2.1 Study design and data source

A cross-sectional study was conducted using the Mexican Family Life Survey (MxFLS). The MxFLS, a representative survey of the Mexican population at the national, urban, rural, and regional levels [21], is a face-to-face in-home survey that collects extensive information at the household and individual levels, including anthropometric measures of all household members and intertemporal preferences of adults, which is conducted every six years. Specifically, it obtained information about intertemporal preferences, the obesity status of parents or caregivers and children, and information about other individual and household characteristics and lifestyle habits. Data from the 2012 MxFLS was used in these analyses, as the data from the 2018 survey was not released at the time of writing.

### 2.2 Study population

The analysis included children between 2–17 years old and adults (≥18 years and older) taking care of at least one of the included children. The MxFLS captures who is the main caregiver for the children, which is either a parent or another family member (e.g., grandparent, uncles/aunts). If this information was not provided, or if the main reported caregiver did not live in the same house as the children (i.e., if the children’s caregiver is an employee), the information from one of the parents living in the household was used. Hereafter, we use the term "caregivers" to relate to parents or other family members helping with childcare. Participants with complete information on time preferences and other covariables described below were included.

### 2.3 Dependent variable

The primary dependent variable was based on children’s body mass index (BMI, [kg/m^2^]). Both height and weight were measured by trained personnel, using standardized instruments, at home when carrying out the survey [21]. Child overweight and obesity were defined using the World Health Organization (WHO) BMI cut-points [22]. Child BMI z-scores were calculated based on age and sex using the recommended WHO growth standards for children aged 2 to 4 years and the WHO reference data for children aged 5–17. Children were divided into three categories: normal weight (for ages two to five, BMI z-scores <2; and older than five, z-scores <1), overweight (for ages two and five, 2≤ BMI z-score <3; and older than five, 1≤ BMI z-score <2), and obesity (for age two to five, BMI z-score ≥3; and older than five, BMI z-score ≥2) [22]. Few children were underweight; thus, this group was combined with the normal-weight children for this study.

### 2.4 Independent variables

The primary independent variables were the caregivers’ time preferences which were defined by two concepts: patience and time consistency. Patience refers to the extent an individual is willing to wait to receive a higher reward in the future, compared to a smaller immediate reward. Consistent with previous studies [3, 12, 23], patience was expressed in terms of patient, medium patient, and impatient. Time consistency refers to how individuals modify their willingness to wait when the time frame changes. Aligned with the literature, time consistency was expressed in terms of time consistent, present bias, and future bias [5, 17].

**Fig 1:**
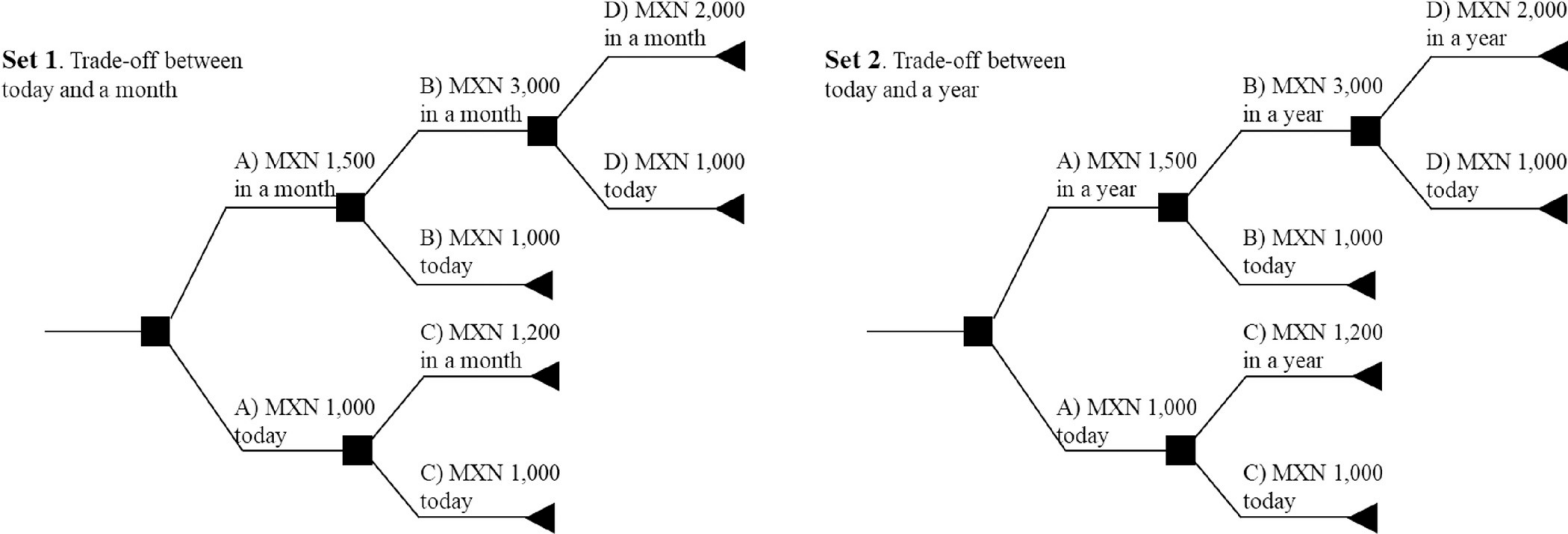
Survey’s trade-off to measure time preferences.

Fig 1 presents the trade-offs and rewards used to construct time preference measures in the MxFLS. Adult participants were asked two sets of questions. In the first set of questions, adults were asked to choose between 1,000 Mexican pesos (MXN) (approximately $50) today or MXN 1,500 (approximately USD) in a month; if they chose MXN 1,500 in a month over 1,000 today, they were asked if they would choose MXN 3,000 in a month or MXN 1,000 today (option B in Fig 1). If the answer was MXN 3,000, participants were asked whether they preferred MXN 2,000 in a month or MXN 1,000 today (option C in Fig 1). On the other hand, if participants chose MXN 1,000 today instead of MXN 1,500 in a month, participants were asked to choose between MXN 1,200 in a month or MXN 1,000 today (option D in Fig 1), and the survey ended. In addition to asking to choose a reward now or in a month, the second set of questions asked participants to decide between today and in a year similarly. These two questions were used to estimate the parameters of patience (represented by δ) and time consistency (β).

Therefore, using the survey information in the preference section and following previous literature on time preferences, as well as previously published thresholds to determine patience and time-consistency parameters [3, 10, 17], the first set of questions of the survey (select a hypothetical amount to receive today versus a possible larger sum in a month) was employed to calculate patience (i.e., δ) as:

$$\delta =\frac{\$1,000}{minimum\;amount\;of\;money\;willing\;to\;accept\;in\;a\;month\;over\;\$1,000\;today}$$

Consistent with previous works [3, 10, 17], this information was used to categorize the children’s caregivers as patient if the children’s caregiver was willing to accept MXN 1,200 in a month instead of MXN 1,000 today (δ = 0.83 = $1000/1,200) and impatient when they preferred $1,000 today than $3,000 in a month (δ = 0.33; $1,000/$3,000). Those caregivers preferring to accept MXN 1,500 (δ = 0.67) or $2,000 in a month (δ = 0.50) instead of MXN 1,000 today were considered medium patient. Children’s caregivers who always chose to receive MXN 1,000 today (δ = 1) regardless of the hypothetical amount in a month were considered impatient, assuming there was no quantity higher enough to make them willing to defer their reward.

Similarly, we calculated the parameter for time consistency based on the responses from both sets of questions:

$$\beta =\frac{minimum\;amount\;of\;money\;willing\;to\;accept\;in\;a\;month\;over\;\$1,000\;today}{\;minimum\;amount\;of\;money\;willing\;to\;accept\;in\;one\;year\;over\;\$1,000\;today}$$

Children’s caregivers were classified as present biased if the amount of money they chose in a shorter term was higher than in the future (β>1, i.e., MXN 3,000 in a month and MXN 2,000 in a year), future biased if long term amount was higher (β<1, i.e., MXN 1,500 in a month and MXN 2,000 in a year), or time consistent if they chose the same amount in both 1-month and 1-year periods (β = 1). The methods used to derive the thresholds for δ and β used in this study were consistent with previous works [3, 10, 17].

Children were described in terms of age groups (i.e., 2–7, 8–12, and 13 years and older), sex, weekly hours spent in front of a screen for other proposes than schoolwork, and weekly hours allocated to physical activity. Caregivers were also described in terms of age groups (i.e., 18–39, 40 years or older), sex, education (i.e., less than high school, high school and more), marital status (single or married) and BMI categories (i.e., normal weight: BMI<25 kg/m^2^, overweight: 25≤BMI<30 kg/m^2^, and obesity BMI≥30 kg/m^2^). The households in which the children lived were described in terms of socioeconomic status (SES) stratified as low, medium, and high, constructed through principal component analysis with a combination of household assets and income data [24], and household location identified as rural/urban status.

### 2.5 Statistical analysis

Mean, standard deviations (S.D.s), and percentages for continuous and discrete variables, respectively, were first used to describe the populations. The children and the adult populations were stratified by BMI categories to identify any differences in baseline characteristics. To further describe the data, caregivers’ time preferences, patience, and time consistency were presented by parents’ BMI categories, education, household SES, and location. Multiple pairwise comparisons (Tukey’s test) for continuous variables [25] and an adaptation of Tukey’s test for discrete variables [26] were used to test for any differences between BMI categories.

To evaluate the association between caregivers’ time preferences and children’s overweight and obesity status, we used multivariable multinomial logistic regressions and reported odds ratios (OR) and 95% confidence intervals (CI) using a two-step strategy. First, to provide a baseline association between caregiver’s time preferences and children’s obesity, we adjusted for children’s age groups and sex and caregiver’s age group, sex, marital status and education, and household SES. In a second step, we ran another model in which we added the caregiver’s BMI and the weekly number of hours children spent in front of a screen or doing physical activity. Correlation and collinearity between covariates used in the model were tested using Pearson correlations and Variance Inflation Factors (VIF), respectively. To deal with potential measurement errors associated with caregivers’ BMI, we also ran a model in which caregivers’ BMI was analyzed as a continuous polynomial variable. Since the association between parental time preferences and children’s obesity status may also depend on children’s sex and age, separate stratified models were conducted by these two variables.

While multinomial logistic models are commonly used to analyze BMI categories [27, 28], a sensitivity analysis was conducted by analyzing the data using a logistic regression model with normal weight as the comparison group as it was done in the U.S. study on parental time preferences [17] as well as a generalized ordinal models (gologit). Gologit models are different from multinomial logistic models as they modify the comparison group allowing comparisons to all categories greater than the current to all those less than or equal to the current one (i.e., normal weight versus overweight and obesity; normal weight and overweight versus obesity). These models have been recently used to model ordinal data in the areas of multidrug resistance [29], tobacco or opioid consumption [30, 31], and depression or pain control [32, 33].

Sampling weights were used for all descriptive and models estimations per the MxFLS survey instructions [21]. In addition, when calculating the 95% C.I.s associated with the O.R.s, the standard errors were clustered at the household level as some children have the same caregiver and share the same household. All analyses were conducted using Stata SE 16.1.

## 3. Results

### 3.1 Study population

From the original sample of 12,089 children with information about their caregiver, 1,833 did not have complete anthropometric information, 240 missed the time allocation variable, 342 did not have information about household characteristics, and 572 did not have information about the caregiver’s time preferences. S1 Appendix presents this information. The analysis included 9,102 children between two and 17 years of age, representing more than 13 million Mexican children and adolescents when the survey results were extrapolated to the Mexican population.

The mean age of the children was almost 10 (SD = 5), 49% were female, and children spent almost 14 hours (SD = 15) per week in front of a screen and 13 (SD = 13) hours per week of physical activity. The distribution of BMI categories indicated that 70% of the children had normal weight, 18% were classified as overweight, and 12% had obesity. Older children and males were statistically significantly more likely to have overweight or obesity relative to younger children and females. Similarly, children with overweight or obesity spent statistically significantly less time physically active and more time on a screen than children with normal weight. Table 1 provides the details.

**Table 1:** Descriptive statistics for Mexican children between 2–17 years of age and by BMI category.

	Total	Normal weight	Overweight	Obesity
**Sample size, %**	9,102	6,418, 70%	1,567–18%	1,117–12%
**[weighted population]**	[13,752,356]	[9,235,645]	[2,565,12]	[1,951,699]
**Age (years)** ^a, b^	** **			
*Mean (Std*. *Dev)*	9.58 (4.7)	8.97 (4.8)	10.98 (4.6)	10.62 (4.3)
By group (%)				
2–7 ^a, b^	37	44	21	18
8–12 ^a, b, c^	32	29	40	47
13–17 ^a, c^	31	27	39	35
**Sex**				
Female (%) ^b, c^	49	49	53	40
**Physical activity**	** **			
(Hours per week) ^a, b^				
*Mean (Std*. *Dev)*	13.94 (14.8)	14.76 (11.6)	12.44 (11.6)	11.90 (11)
**Screen time**	** **			
(Hours per week) ^a, b^				
*Mean (Std*. *Dev)*	12.74 (12.8)	12.01 (16.9)	14.34 (13.8)	14.23 (13.7)

The survey design was considered for all estimations. a, b, c, d indicates statistical significance (i.e., P< 0.05) for the following comparisons: a: normal weight vs. overweight, b: normal weight vs. obesity, c: overweight vs. obesity. Source: authors construction with information from Mexican Family Survey Life [21]

The baseline characteristics of the children’s caregivers (i.e., n = 5,842 representing more than 9.5 million Mexican caregivers) are presented in Table 2 for the overall population and stratified by children’s BMI status. Approximately 94% of the caregivers were the children’s parents, while the remaining 6% were family members. The mean age of the caregivers was 37 (SD = 11) years; 95% were female, 81% had a lower education than high school, and 87% were married. Based on caregiver BMI, 38% were classified as overweight, and 37% had obesity. Almost two-thirds (61%) of the caregivers lived in an urban setting, and more than half were in a medium SES household (56%). The stratified analyses revealed several differences in the caregivers’ characteristics by children’s BMI category. For example, caregivers of normal-weight children were younger (68%) than children with overweight (64%) or obesity (59%) (p<0.01 for all comparisons between BMI categories). Children with overweight (45%) or obesity (58%) were also more likely to have caregivers with obesity compared to children with normal weight (31%; p<0.01 for all comparisons). Caregivers living in high SES neighborhoods or urban settings were also more likely to have children with obesity. Table 2 presents the details.

**Table 2:** Descriptive statistics for Mexican caregivers and per by BMI category of children.

Caregiver characteristics		Children’s BMI status		
	Total	Normal weight	Overweight	Obesity
Sample size caregivers [weighted population]	5,842	4,439, 76%	876, 15%	526, 9%
	[9,585,138]	[7,284,705]	[1,437,771]	[862,662]
Caregiver Characteristics				
**Age (years)** ^a, b^	** **			
*Mean (Std*. *Dev)*	37.29 (10.8)	36.20 (10.2)	38.43 (9.2)	38.24 (9.3)
18–39 (%) ^a, b^	66	68	64	59
**Sex (%)**				
Female	95	95	97	96
**Schooling (%)**				
Less than high school ^b^	81	82	78	79
**Marital status (%)**				
Married	87	85	90	90
**BMI category (%)**				
Normal weight ^a, b, c^	25	31	17	13
Overweight ^b, c^	38	38	38	29
Obesity ^a, b, c^	37	31	45	58
**Household characteristics**				
**Socioeconomic status (%)**	** **			
Low ^a, b^	16	17	14	9
Medium	56	56	57	55
High ^a, b^	29	27	29	36
**Location (%)**				
Urban ^a, b^	61	59	66	66

The survey design was considered for all estimations. a, b, c, d indicates statistical significance (i.e., P< 0.05) for the following comparisons: a: normal weight vs. overweight, b: normal weight vs. obesity, c: overweight vs. obesity. Source: authors construction with information from Mexican Family Survey Life [21]

### 3.2 Caregiver’s time preferences

Table 3 presents the children caregivers’ time preferences for the overall population and when the caregivers were stratified by their BMI categories, household characteristics, and children’s physical activity and screen time. Overall, 67% of the caregivers’ population was impatient (i.e., preferred to have an immediate reward than a larger one in the future), and 14% were patient, the remaining 19% being medium patient. Between 6% (1-month assessment) and 7% (1 year) of caregivers chose the upper value of MXN 3,000 over MXN 1,000 today (Table A1 in S1 Appendix). More than half of the caregivers (58%) were time consistent (i.e., choices did not change when considering future regards in a month or one year), and 31% had a future bias. Approximately 82% of impatient caregivers were also time-consistent (Table A2 in S1 Appendix). The stratified analyses indicated differences in caregivers’ time preferences based on their characteristics. For example, patient caregivers were more likely to live in high SES neighborhoods (i.e., 43% versus 25%; p < .005) or urban areas (68% versus 60%, p<0.05) than impatient caregivers. Caregivers with a future bias were statistically significantly more likely to live in high SES neighborhoods (39%) or in urban areas (68%) than caregivers who were time consistent (25% for high SES and 60% for urban areas) or had a present bias (32% for high SES and 55% for urban area). Other statistically significant differences were also seen in caregivers’ BMI categories or children’s time spent doing physical activities or on a screen (Table 3).

**Table 3:** Caregiver time preferences by BMI categories, household characteristics, physical activity and screen time.

		Patience			Time consistency	
	Patient	Medium patient	Impatient	Time consistent	Present bias	Future Bias
Caregiver variables						
**All**	14	19	67	58	11	31
**Caregiver BMI categories (%)**						
Normal weight	26	23	26	24	31	24
Overweight ^d^	39	43	36	37	39	39
Obesity ^d^	35	34	38	39	30	37
**Household characteristics **						
**Socioeconomic status (%)**						
Low ^h^	14	10	19	19	17	10
Medium ^e, f, g^	53	53	56	56	51	51
High ^e, f, g, h^	43	37	25	25	32	39
**Location**						
Urban ^h, i,^	68	67	60	60	55	68
**Children’s time allocation**						
**Physical activity (%)**						
Mean (std dev) ^e^	14.97 (16.0)	13.63 (16.4)	14.90 (15.9)	14.11 (16.1)	16.78 (15.2)	14.83 (15.4)
**Screen time (%)**						
Mean (std dev) ^e^	15.27 (11.6)	13.88 (11.5)	12.46 (12.6)	12.34 (11.6)	13.35 (11.9)	14.50 (11.5)

Survey design is considered for all estimations. f-m indicates statistical significance (i.e., P< 0.05); d: patient vs. medium patient, e: patient vs. impatient, f: medium patient vs. impatient, g: time consistent vs. present bias, h: time consistent vs. future bias, i: present bias vs. future bias. Source: authors construction with information from [21]

### 3.3 Association between caregivers’ time preferences and children’s BMI status

The results of the multivariable multinomial logistic regressions are provided in Table 4 for the overall population of children and according to the two models (Model 1 without caregiver’s BMI and children’s time spent on T.V. or physical activity, and Model 2 including all variables). There was no evidence of correlation or multicollinearity between the covariates (all correlations below 0.35 and an overall VIF = 1.12). Having a caregiver who was classified as impatient, compared to patient, was not significantly associated with increased odds of childhood overweight or obesity. According to Model 1, having a medium patient caregiver, compared to patient, was significantly associated with increased odds of overweight in children (OR 1.47; 95% CI: 1.01, 2.14). In terms of time consistency, caregivers with a present (OR: 2.26; 95% CI: 1.36, 3.73) or future (OR: 1.45; 95% CI: 1.06, 1.99) bias, compared to caregivers who were time consistent in their choices, were also more likely to have children with obesity. As shown in Table 4, the overall time preference results did not change when the caregiver’s BMI and children’s time spent on T.V. or physical activity were included, although the magnitude of the O.R.s changed slightly. For example, for the association between having a medium patient caregiver and overweight, the OR changed from 1.47 (95% CI: 1.01, 2.14) to 1.74 (95% CI: 1.19, 2.53). For a present biased caregiver, the OR changed from 2.26 (95% CI: 1.36, 3.73) to 2.52 (95% CI: 1.74, 3.66). However, the caregiver’s BMI and time spent on T.V. or physical activity were independently associated with children’s obesity.

**Table 4:** Multinomial logistic model for children obesity status, caregiver’s patience level, and time-consistency. Overall and stepwise analysis.

		Without caregiver BMI, Physical activity, screen time		All children. Complete model	
Odds Ratio		Overweight	Obesity	Overweight	Obesity
(Normal weight as reference)					
**Caregiver’s patience**					
**(reference: patient)**					
Medium patience		1.47*	0.98	1.74**	1
		[1.01–2.14]	[0.62–1.53]	[1.19–2.53]	[0.54–1.83]
Impatient		1.12	1.40+	1.21	1.30+
		[0.83–1.52]	[0.95–1.93]	[0.80–1.81]	[0.97–1.73]
**Caregiver’s time consistency**					
**(Reference: Time consistent)**					
Present bias		1.04	2.26**	1.11	2.52**
		[0.79–1.37]	[1.36–3.73]	[0.73–1.69]	[1.74–3.66]
Future bias		1.13	1.45*	1.23	1.48**
		[0.87–1.47]	[1.06–1.99]	[0.85–1.77]	[1.11–1.97]
**Caregiver’s BMI**					
**(Reference: Normal weight)**					
Overweight				2.06**	1.70**
				[1.50–2.85]	[1.38–2.10]
Obesity				2.71**	4.03**
				[2.09–3.52]	[2.88–5.63]
**Caregiver’s age group**					
**(reference 18–39)**					
40 and older		0.87	1.16	0.79	0.98
		[0.69–1.10]	[0.95–1.41]	[0.56–1.12]	[0.79–1.22]
**Caregiver’s sex**					
**(Reference: female)**					
Male		1.09	0.98	1.14	0.98
		[0.56–2.11]	[0.57–1.68]	[0.57–2.28]	[0.58–1.67]
**Caregiver’s marital status**					
**(Reference: Married)**					
Single		0.64*	0.62	0.59+	0.46
		[0.43–0.95]	[0.30–1.28]	[0.34–1.04]	[0.16–1.27]
**Caregiver’s schooling level**					
**(Reference: Less than high school)**					
High School & more		1.31	1.21**	1.31	1.15**
		[0.93–1.84]	[1.09–1.34]	[0.88–1.94]	[1.06–1.26]
**Household socioeconomic status**					
**(reference: Low)**					
Medium		1.08	1.74**		
		[0.74–1.57]	[1.24–2.43]	0.81+	1.2
High		1.11	2.33**	[0.64–1.04]	[0.88–1.65]
		[0.81–1.53]	[1.73–3.14]	0.87	1.75*
**Household location: Urban/rural**				[0.59–1.30]	[1.15–2.65]
**(Reference: Rural)**					
Urban		1.35**	1.35**	1.44**	1.27*
		[1.20–1.53]	[1.20–1.53]	[1.28–1.63]	[1.05–1.54]
**Child’s age group (reference: 2–7)**					
08–12		3.29**	4.19**	3.04**	3.78**
		[2.33–4.65]	[3.32–5.30]	[2.17–4.26]	[3.00–4.77]
13–17		3.28**	3.00**	2.69**	2.87**
		[2.75–3.91]	[1.95–4.59]	[1.96–3.69]	[2.00–4.10]
**Child’s sex (reference: Female)**					
Male		0.87	1.44*	1.05	1.65**
		[0.60–1.26]	[1.01–2.05]	[0.88–1.26]	[1.20–2.26]
**Child’s physical activity (hours per week)**				0.98**	0.98**
				[0.98–0.99]	[0.98–0.99]
**Child’s Screen time (hours per week)**				1.02**	1.02**
				[1.01–1.02]	[1.01–1.03]
**Constant**		0.08**	0.02**	0.05**	0.01**
		[0.06–0.12]	[0.01–0.03]	[0.03–0.08]	[0.01–0.02]
**Observations**		9,102		9,102	
*Expanded to*		*[13,752,356]*		*[13,752,356]*	

Considering the low number of male caregivers (N = 386) and the fact that the caregiver’s sex was not associated with children’s obesity (Table 4), we excluded the caregiver’s sex in our stratified analyses. However, we included the caregiver’s BMI and children’s time spent watching T.V. or doing physical activity. Also shown in Table 5, our stratified analyses suggested that the association between time preferences and children’s obesity status may have been modified by sex or age. Our estimates, however, were not precisely estimated (the confidence intervals were wide and overlapped across almost all comparisons). For example, having a caregiver impatient increased the odds of having obesity in children 13–17 years of age by 2.48 (95% CI: 1.60, 3.84) compared to 1.11 (95% CI: 0.68, 1.82) in children 8–12 years of age. Similarly, having a caregiver with a future bias increased the odds of having obesity in male children (OR: 2.11; 95% CI: 1.21, 3.69) while it decreased the odds of having obesity in female children (OR: 0.96; 95% CI: 0.72, 1.29).

**Table 5:** Multinomial logistic model for children obesity status, caregiver’s patience level, and time-consistency. Stratified analysis by children’s sex and age.

	2–7 years old		8–12 years old		13–17 years old		Female		Male	
Odds Ratio	Overweight	Obesity	Overweight	Obesity	Overweight	Obesity	Overweight	Obesity	Overweight	Obesity
(Normal weight as reference)										
**Caregiver’s patience**										
**(reference: patient)**										
Medium patience	1.12	1.37	2.82*	0.73	0.76	1.07	1.67	1.2	1.81*	0.83
	[0.63–1.97]	[0.85–2.20]	[1.62–4.92]	[0.24–2.23]	[0.52–1.12]	[0.45–2.58]	[0.86–3.21]	[0.81–1.78]	[1.05–3.13]	[0.38–1.79]
Impatient	0.78	1.09	1.83*	1.11	0.66	2.48*	1.11	1.06	1.28	1.40*
	[0.48–1.25]	[0.49–2.44]	[1.12–2.99]	[0.68–1.82]	[0.32–1.35]	[1.60–3.84]	[0.55–2.24]	[0.68–1.66]	[0.85–1.94]	[1.15–1.71]
**Caregiver’s time consistency**										
**(Reference: Time consistent)**										
Present bias	0.79	1.66	1.84*	1.73	0.77	8.27*	1.4	2.89*	0.89	2.44*
	[0.34–1.84]	[0.77–3.58]	[1.05–3.23]	[0.83–3.60]	[0.16–3.68]	[4.95–13.83]	[0.92–2.12]	[1.46–5.72]	[0.56–1.41]	[1.10–5.41]
Future bias	1.07	1.05	1.89*	1.65*	0.84	3.15*	1.32	0.96	1.2	2.11*
	[0.52–2.19]	[0.59–1.87]	[1.59–2.24]	[1.22–2.22]	[0.35–2.02]	[2.26–4.38]	[0.82–2.14]	[0.72–1.29]	[0.79–1.82]	[1.21–3.69]
**Caregiver’s BMI**										
**(Reference: Normal weight)**										
Overweight	2.52*	1.41	1.78*	1.60	2.03*	1.93*	2.39*	2.72*	1.96*	1.46*
	[1.42–4.50]	[0.74–2.68]	[1.31–2.42]	[0.95–2.69]	[1.43–2.87]	[1.32–2.82]	[1.35–4.24]	[1.39–5.34]	[1.55–2.49]	[1.13–1.90]
Obesity	2.85*	4.22*	2.89*	3.46*	2.55*	5.54*	2.89*	8.68*	2.74*	2.89*
	[1.73–4.68]	[1.77–10.08]	[2.10–3.98]	[2.25–5.34]	[1.83–3.54]	[3.01–10.18]	[1.85–4.53]	[4.64–16.23]	[1.69–4.43]	[1.92–4.36]
**Caregiver’s age group**										
**(reference 18–39)**										
40 and older	1.17	1.39	0.68*	0.99	0.86	0.71*	0.83	1.23	0.81	0.88
	[0.79–1.74]	[0.79–2.45]	[0.48–0.97]	[0.66–1.47]	[0.65–1.13]	[0.52–0.96]	[0.63–1.09]	[0.98–1.53]	[0.51–1.29]	[0.63–1.21]
**Caregiver’s marital status**										
**(Reference: Married)**										
Single	1.07	0.39	0.58	0.41	0.33*	0.61	0.56*	0.32*	0.6	0.56
	[0.49–2.38]	[0.14–1.06]	[0.27–1.24]	[0.12–1.34]	[0.14–0.78]	[0.21–1.82]	[0.32–0.99]	[0.11–0.95]	[0.21–1.67]	[0.18–1.73]
**Caregiver’s schooling level**										
**(Reference: Less than high school)**										
High School & more	1.09	0.92	2.06*	1.82*	0.82	0.56	0.9	0.88	1.77	1.45*
	[0.69–1.72]	[0.47–1.80]	[1.01–4.20]	[1.40–2.36]	[0.45–1.52]	[0.25–1.25]	[0.68–1.19]	[0.48–1.61]	[0.93–3.39]	[1.00–2.10]
**Household socioeconomic status**										
**(reference: Low)**										
Medium	0.98	0.88	0.57*	0.95	1.39	4.81*	0.73	0.72	0.83	1.48*
	[0.54–1.79]	[0.52–1.48]	[0.38–0.84]	[0.55–1.66]	[0.72–2.67]	[2.62–8.84]	[0.46–1.16]	[0.41–1.30]	[0.64–1.08]	[1.12–1.97]
High	0.98	1.88*	0.65	1.14	1.31	7.58*	1.09	1.51	0.67	1.78*
	[0.47–2.07]	[1.31–2.72]	[0.34–1.24]	[0.54–2.41]	[0.45–3.81]	[4.12–13.93]	[0.64–1.85]	[0.72–3.16]	[0.44–1.02]	[1.20–2.65]
**Household location: Urban/rural**										
**(Reference: Rural)**										
Urban	1.66*	1.46*	1.31*	1.27	1.46*	0.81	1.37*	1.31	1.55*	1.28*
	[1.11–2.48]	[1.04–2.05]	[1.13–1.52]	[1.00–1.62]	[1.08–1.97]	[0.61–1.07]	[1.15–1.63]	[0.91–1.87]	[1.34–1.80]	[1.02–1.61]
**Child’s age group (reference: 2–7)**										
08–12							3.81*	3.23*	2.51*	4.21*
							[2.34–6.19]	[2.62–3.98]	[1.80–3.49]	[3.06–5.80]
13–17							3.41*	2.81*	2.16*	2.78*
							[2.21–5.28]	[1.86–4.24]	[1.55–3.01]	[1.38–5.62]
**Child’s sex (reference: Female)**										
Male	1.60*	1.79*	0.89	1.73*	0.94	1.29				
	[1.08–2.37]	[1.33–2.42]	[0.65–1.21]	[1.20–2.50]	[0.57–1.53]	[0.68–2.43]				
**Child’s physical activity (hours per week)**	0.98*	0.99*	0.99	0.98*	0.96	0.96*	0.98*	0.97*	0.99*	0.99*
	[0.97–1.00]	[0.98–1.00]	[0.97–1.00]	[0.97–1.00]	[0.91–1.01]	[0.95–0.97]	[0.96–0.99]	[0.96–0.98]	[0.98–1.00]	[0.98–1.00]
**Child’s Screen time (hours per week)**	1.02*	1.02	1	1.01	1.04*	1.04*	1.03*	1.04*	1.01	1
	[1.00–1.04]	[0.99–1.05]	[0.99–1.02]	[0.99–1.04]	[1.03–1.05]	[1.02–1.05]	[1.01–1.04]	[1.03–1.05]	[1.00–1.02]	[0.98–1.02]
**Constant**	0.04*	0.01*	0.14*	0.08*	0.19*	0.01*	0.04*	0.01*	0.05*	0.02*
	[0.02–0.07]	[0.00–0.05]	[0.09–0.23]	[0.05–0.15]	[0.06–0.61]	[0.00–0.01]	[0.01–0.11]	[0.00–0.03]	[0.03–0.09]	[0.01–0.03]
**Observations**	3,496		2,699		2,907		4,476		4,626	
*Expanded to*	*[4,844,922*]		*[4,350,232] *		*[4,22,960]*		*[6,486,408]*		*[6,912,935]*	

Notes: ciEform in brackets.

* p<0.05. Survey design considered for estimations. Standard Errors are adjusted at the household level. ~For purposes other than schoolwork.

### 3.4 Other associations with children BMI status

As shown in Table 4, several other variables were also significantly associated with children’s BMI categories in the main analyses or the stratified analyses by children’s age groups and sex. Having a caregiver with overweight (except for children 8–12 years of age with obesity) or with obesity increased the odds of overweight or obesity in children compared to having a caregiver with normal weight in all analyses (i.e., females with overweight OR: 2.39; 95% CI: 1.35, 4.24 and males with overweight OR:1.96; 95% CI: 1.55, 2.49). Except for female children with obesity, living in rural areas also significantly increased the odds of overweight or obesity in children. Several other variables were associated with children’s BMI status, but the association depended on the studied populations rather than being seen across all analyses.

### 3.5 Sensitivity analyses

The results of the gologit model and the logistic model were mostly aligned with the multinomial logistic regression model regarding the association between time preferences and caregiver’s obesity status and children’s BMI status (see Table 6). For example, the multinomial model displayed an OR of 2.52 (95% CI:1.72, 3.70) for a present bias caregiver and a child with overweight, and the generalized ordinal model showed a coefficient of 2.42 (95% CI: 1.85, 3.17) and 1.64 (95% CI: 1.21, 2.23) in the logistic model. Differences were found in the association with an urban setting losing significance in the gologit model and in some magnitude of coefficients (but with overlapping C.I.s), i.e., having a caregiver with obesity with an OR of 4.03 (95% CI:2.90, 5.59) in the multinomial model, and 3.27 (95% CI:2.59, 4.13) and 3.23 (95% CI: 2.56, 4.07) in the generalized ordinal model, and the logistic model, respectively. The association of the other independent variables with children’s BMI status for the overall population or the stratified analyses was also consistent with the results from the multinomial logistic model (see S1 Appendix).

**Table 6:** Generalized ordinal logistic and logistic model for children obesity status, caregiver’s patience level, and time-consistency.

	GOLOGIT		Logit
Odds Ratio	Overweight and obesity	Obesity	Overweight and obesity
**Caregiver’s patience (reference: patient)**			
Medium patience	1.37	0.89	1.4
	[0.86–2.20]	[0.53–1.49]	[0.90–2.19]
Impatient	1.27	1.27	1.25
	[0.90–1.80]	[0.90–1.80]	[0.90–1.76]
**Caregiver’s time consistency (reference: Time consistent)**			
Present bias	1.70*	2.42*	1.64*
	[1.26–2.30]	[1.85–3.17]	[1.21–2.23]
Future bias	1.39*	1.39*	1.34*
	[1.18–1.64]	[1.18–1.64]	[1.10–1.63]
**Caregiver’s obesity status (reference: Normal weight)**			
Overweight	1.86*	1.86*	1.92*
	[1.53–2.27]	[1.53–2.27]	[1.53–2.41]
Obesity	3.27*	3.27*	3.23*
	[2.59–4.13]	[2.59–4.13]	[2.56–4.07]
**Caregiver’s age group (reference 18–39)**			
40 and older	0.9	0.9	0.87
	[0.73–1.12]	[0.73–1.12]	[0.68–1.12]
**Caregiver’s marital Status (reference: Married)**			
Single	0.56	0.56	0.54
	[0.26–1.21]	[0.26–1.21]	[0.27–1.10]
**Caregiver’s schooling level (reference: Less than high school)**			
High School & more	1.20	1.20	1.25
	[0.98–1.47]	[0.98–1.47]	[0.96–1.62]
**Household socioeconomic status (reference: Low)**			
Medium	1.01	1.01	0.94
	[0.82–1.24]	[0.82–1.24]	[0.75–1.18]
High	1.27	1.27	1.15
	[0.91–1.79]	[0.91–1.79]	[0.80–1.67]
**Household location: Urban/rural (reference: Rural)**			
Urban	1.33*	1.14	1.36*
	[1.16–1.54]	[0.98–1.32]	[1.20–1.54]
**Child’s age group (reference: 2–7)**			
8–12	3.22*	3.22*	3.31*
	[2.68–3.86]	[2.68–3.86]	[2.69–4.09]
13–17	2.61*	2.61*	2.72*
	[1.94–3.50]	[1.94–3.50]	[1.96–3.78]
**Child’s sex (reference: Female)**			
Male	1.25*	1.67*	1.27*
	[1.03–1.52]	[1.26–2.20]	[1.03–1.56]
**Child’s physical activity (hours per week)**	0.98*	0.98*	0.98*
	[0.98–0.99]	[0.98–0.99]	[0.98–0.99]
**Child’s Screen time (hours per week)**	1.02*	1.02*	1.02*
	[1.01–1.02]	[1.01–1.02]	[1.01–1.03]
**Constant**	0.05*	0.02*	0.06*
	[0.04–0.08]	[0.01–0.02]	[0.04–0.08]
**Observations**	9,102		9,102
*Expanded to*	*[13,752,356]*		*[13,752,356]*

Notes: ciEform in brackets.

* p<0.05. Survey design considered for estimations. Standard Errors are adjusted at the household level. ~For purposes other than schoolwork.

Our sensitivity analyses to deal with potential measurement error with caregivers’ BMI showed that modeling caregivers’ BMI as a continuous polynomial variable did not change the results (see Table A4 in S1 Appendix). Furthermore, only first-degree BMI was significant (for children obesity OR: 1.24, 95% CI: 1.07, 1.43).

## 4. Discussion

To the best of our knowledge, this is only the second study evaluating the association between caregivers’ time preferences and overweight and obesity in children and the first study conducted in Latin America or exploring the association of caregivers’ time preferences and children’s BMI status by children age groups and sex. Based on the results of a survey representative of the Mexican population, our findings indicated that more than two-thirds of children’s caregivers were impatient, while approximately 60% were time consistent in their choices. Patient and time-consistent adults have been shown to make healthier choices [3, 10, 17, 34] than impatient or time-inconsistent adults.

The results of our analysis suggest that children caregivers’ time preferences were associated with children’s BMI status; in particular, present or future-biased time preferences were associated with increased odds of childhood overweight or obesity. Present-biased caregivers prefer to receive a larger reward in the short term than a higher reward in the future, which could lead to unhealthy behaviors for themselves and their children [17, 35], including the availability of unhealthy food at home and the lack of promotion of physical activity. While the relationship between future-biased caregivers and children’s obesity status is less intuitive, few studies have investigated the effects of future bias on healthy decisions [17, 36]. Some experiments have shown that the individual’s willingness to get a larger reward in the future evolves over time [13, 36, 37], which could explain some of the results as a future-biased individual at time t may become a present bias individually at time t+1. It is also possible that caregivers value having a bigger child, and child overweight or obesity may not be perceived as a concern. Future research should examine the reasons for a potential association between future biased time preferences and obesity.

Caregiver time preferences also showed increasing odds with children’s age which can be explained by imitation of unhealthy parental behavior–same diet and exercise routines [38, 39]. It should also be noted that in Mexico, sales of junk food are forbidden in elementary schools or nearby, while daily physical activity is only mandatory in elementary schools [40]. Compared to younger children attending elementary schools, Mexican children of middle school age are exposed to a more obesogenic environment (unhealthy food choices; lack of mandatory physician activity), which could also explain the age pattern in our study. Similarly, the obesity status of the caregivers was associated with the children’s BMI status, which may be explained by straightforward genetics, the imitation of food patterns by children, higher availability of high-dense energy food at home, or lower promotion for physical activity [41–44]. Living in an urban area was also associated with children’s BMI status [45, 46].

When comparing our results with the previous literature, BMI categories’ prevalence for children and their caregivers was consistent with official reports in Mexico for 2012 [47]. Similarly, our household characteristics (educational and SES status, rural status) were also aligned with nationally representative Mexican data [48]. It is not easy to compare our results with the only published study on parental time preferences and children’s BMI status due to important cultural and economic differences between the U.S. and Mexico. While the U.S. study found an association between patience and children’s obesity status [17], our results did not support this association. This difference could be explained because we found a higher proportion of impatient adults in Mexico than in the U.S. (66% in our study versus 27% in the U.S. [17]). Differences between countries in adults’ time preferences have been previously reported, showing a lower patience level in highly religious countries, middle-income, and with higher agricultural dependence [49]. Also, women were reported to be more impatient than men [49], which were the main caregivers in this study. Our results on the association of caregivers’ BMI status and children’s BMI were also consistent with several other studies [44, 50–53] conducted in Mexico or elsewhere. One Mexican study reported a strong association between the nutritional status of parents and obesity in children, which may be explained by a combination of genetics, eating patterns, and environment (i.e., sharing a household) [52].

Even though our findings are novel, this study has some limitations. First, the study is cross-sectional; therefore, causality could not be established. Second, caregiver time preferences were based on intertemporal monetary trade-offs rather than using direct food or dietary decisions. However, this method using intertemporal monetary trade-offs has been broadly used for determining time preferences related to eating, smoking, and other health behaviors [3, 54–56]. Third, diet or intensity of physical activity was not considered in our analyses as this information was not documented in the MxFLS. Fourth, there is a potential loss of information using categorical variables instead of continuous variables (i.e., BMI, caregiver age, SES). However, we explored models in which caregivers’ BMI was analyzed as a continuous variable, which did not change the main results. We kept caregivers’ BMI as a categorical variable in our models. Fifth, the analyses were limited by the data captured in the survey. For example, although we had information on the socioeconomic status of the caregivers, we did not have access to income/spending balance or household debt variables which could be related to time preferences. However, studies that have explored the association between household (or personal) debt and obesity have not found an association between these variables [57–59]. Finally, while in theory, caregivers could have chosen an infinite value compared to MXN 1,000 today instead of the upper value of MXN 3,000 used in the survey, the impact of a potential ceiling effect should be minimal as less than 6% (30-day assessment) and 7% (one-year assessment) chose the upper value of MXN 3,000. Despite the limitations, this study provides relevant evidence about the association between caregiver time preferences and children’s BMI status in Mexico, and this study has several strengths. First, a nationally representative sample of the Mexican population was used, so our results should be useful to inform future policies in Mexico. Secondly, several approaches were used for our regression analyses, and the results’ consistency strengthened the study findings. Finally, the study used not only parents but children’s caregivers, who are often the person making decisions when parents are not available.

Worldwide there is increasing research and policymaking promoting healthy lifestyles to reduce the burden of obesity and overweight in children and adolescents [60, 61]. Designing effective policies is crucial to understand how to support individuals to make healthy choices, especially when policies target children. Several studies have shown the importance of incorporating time preferences when designing policies to change health behaviors such as smoking, physical activity, food consumption, and health investment [11, 62–64]. However, very few studies have evaluated the impact of intergenerational time preferences. Our study found that caregiver time preferences were associated with children’s BMI status. However, the association depended on the children’s BMI status (e.g., overweight or obesity), providing new insights which could be used to develop future policies in Mexico or elsewhere. For example, given that more than two-thirds of caregivers in Mexico were impatient (i.e., 67% as shown in this study), policies aimed at reducing childhood obesity should tailor communications by framing short-term instead of long-term outcomes [64] or creating short-term financial incentive-based interventions [5, 12, 63] An example of such a policy is the ’PROGRESA’ program in Mexico which provides monetary incentives in exchange of school attendance and routine G.P. visits for children [65]. These policies should also consider other important factors such as children’s caregiver BMI status or geography (i.e., urban settings), which were also strongly associated with children’s BMI status.

## Supporting information

S1 Appendix (DOCX)
