# Supplementary material for: Association between children’s caregivers time preferences and childhood overweight and obesity in Mexico
Source: PLoS One. 2024 Mar 7;19(3):e0283455. doi: 10.1371/journal.pone.0283455 (PMC10919595; doi:10.1371/journal.pone.0283455)
Supplement: S1 Appendix — (DOCX) [file pone.0283455.s001.docx]

**Appendix**

Figure A1. Sample flow

**Table A1. Minimum accepted in a month/year over 1,000 today ($1,000 are those preferring today)**

|  | Month | Year |
| --- | --- | --- |
| 1000 | 54% | 79% |
| 1200 | 12% | 3% |
| 1500 | 10% | 3% |
| 2000 | 17% | 8% |
| 3000 | 7% | 6% |

**Table A2. Comparison two different timeframes; measuring consistency.**

|  | Time consistent | Present bias |
| --- | --- | --- |
| Patient | 6.97% | 29.92% |
| Medium patience | 20.14% | 8.60% |
| Impatient | 82.34% | 5.74% |

Table A3. Gologit and logit for children obesity status and caregiver’s patience level, and time-consistency. By age group, and by sex.

|  |  | GOLOGIT | | | | | | | | | | Logit | | | | |
| --- | --- | --- | --- | --- | --- | --- | --- | --- | --- | --- | --- | --- | --- | --- | --- | --- |
|  |  | Girls | | Boys | | 2-7 years | | 8-12 years | | 13-17 years | | Girls | Boys | 2-7 years | 8-12 years | 13-17 years |
| **Odds Ratio** | | Overweight and obesity | Obesity | Overweight and obesity | Obesity | Overweight and obesity | Obesity | Overweight and obesity | Obesity | Overweight and obesity | Obesity | Overweight and obesity | Overweight and obesity | Overweight and obesity | Overweight and obesity |  |
| **(Normal weight as reference)** | |  |  |  |  |  |  |  |  |  |  |  |  |  |  |  |
| **Caregiver’s patience** | |  |  |  |  |  |  |  |  |  |  |  |  |  |  |  |
| **(reference: patient)** | |  |  |  |  |  |  |  |  |  |  |  |  |  |  |  |
| Medium patience | | 1.23 | 1.23 | 1.64 | 0.48 | 0.94 | 1.44 | 1.37 | 1.37 | 1.23 | 0.74 | 1.23 | 1.6 | 0.89 | 1.53 | 1.28 |
|  | | [0.78 - 1.95] | [0.78 - 1.95] | [0.73 - 3.70] | [0.19 - 1.21] | [0.54 - 1.61] | [0.91 - 2.27] | [0.97 - 1.94] | [0.97 - 1.94] | [0.78 - 1.94] | [0.39 - 1.40] | [0.76 - 1.98] | [0.74 - 3.45] | [0.53 - 1.51] | [0.96 - 2.44] | [0.82 - 1.98] |
| Impatient | | 0.9 | 0.9 | 1.43 | 1.02 | 1.24 | 2.99* | 1.15 | 1.15 | 1.31* | 1.31* | 0.91 | 1.42 | 1.13 | 1.11 | 1.33* |
|  | | [0.58 - 1.40] | [0.58 - 1.40] | [0.84 - 2.41] | [0.64 - 1.62] | [0.92 - 1.68] | [2.12 - 4.22] | [0.69 - 1.91] | [0.69 - 1.91] | [1.05 - 1.63] | [1.05 - 1.63] | [0.59 - 1.39] | [0.87 - 2.33] | [0.83 - 1.55] | [0.64 - 1.94] | [1.04 - 1.71] |
| **Caregiver’s time consistency** | |  |  |  |  |  |  |  |  |  |  |  |  |  |  |  |
| **(Reference: Time consistent)** | |  |  |  |  |  |  |  |  |  |  |  |  |  |  |  |
| Present bias | | 1.1 | 1.1 | 1.81 | 1.81 | 3.02* | 9.08* | 1.92* | 2.94* | 1.57 | 2.19* | 1.09 | 1.78 | 2.55* | 1.88* | 1.50 |
|  | | [0.60 - 2.04] | [0.60 - 2.04] | [1.00 - 3.27] | [1.00 - 3.27] | [1.43 - 6.36] | [5.81 - 14.21] | [1.19 - 3.10] | [1.33 - 6.50] | [0.96 - 2.55] | [1.29 - 3.70] | [0.59 - 2.01] | [0.96 - 3.32] | [1.15 - 5.63] | [1.11 - 3.18] | [0.95 - 2.36] |
| Future bias | | 1.05 | 1.05 | 1.75* | 1.75* | 1.51 | 3.84* | 1.25 | 1.25 | 1.65* | 1.65* | 1.07 | 1.78* | 1.4 | 1.19 | 1.57* |
|  | | [0.72 - 1.55] | [0.72 - 1.55] | [1.52 - 2.02] | [1.52 - 2.02] | [0.91 - 2.50] | [3.02 - 4.87] | [0.84 - 1.86] | [0.84 - 1.86] | [1.23 - 2.23] | [1.23 - 2.23] | [0.71 - 1.63] | [1.60 - 1.99] | [0.81 - 2.39] | [0.79 - 1.81] | [1.20 - 2.05] |
| **Caregiver’s obesity status** | |  |  |  |  |  |  |  |  |  |  |  |  |  |  |  |
| **(Reference: Normal weight)** | |  |  |  |  |  |  |  |  |  |  |  |  |  |  |  |
| Overweight | | 2.08* | 1.18 | 1.67* | 1.67* | 1.87* | 1.87* | 2.46* | 2.46* | 1.68* | 1.68* | 2.09* | 1.71* | 1.85* | 2.48* | 1.73* |
|  | | [1.36 - 3.20] | [0.65 - 2.13] | [1.33 - 2.09] | [1.33 - 2.09] | [1.56 - 2.24] | [1.56 - 2.24] | [1.77 - 3.42] | [1.77 - 3.42] | [1.34 - 2.11] | [1.34 - 2.11] | [1.35 - 3.23] | [1.43 - 2.06] | [1.54 - 2.22] | [1.68 - 3.67] | [1.39 - 2.16] |
| Obesity | | 3.39* | 3.39* | 3.12* | 3.12* | 3.58* | 3.58* | 4.18* | 7.43* | 2.76* | 2.76* | 3.36* | 3.17* | 3.27* | 4.23* | 2.83* |
|  | | [2.17 - 5.28] | [2.17 - 5.28] | [2.29 - 4.23] | [2.29 - 4.23] | [2.80 - 4.59] | [2.80 - 4.59] | [3.05 - 5.74] | [4.86 - 11.34] | [2.12 - 3.58] | [2.12 - 3.58] | [2.20 - 5.14] | [2.40 - 4.18] | [2.74 - 3.90] | [2.95 - 6.08] | [2.16 - 3.72] |
| **Caregiver’s age group** | |  |  |  |  |  |  |  |  |  |  |  |  |  |  |  |
| **(reference 18-39)** | |  |  |  |  |  |  |  |  |  |  |  |  |  |  |  |
| 40 and older | | 1.25 | 1.25 | 0.85 | 0.85 | 0.87 | 0.87 | 0.98 | 1.27* | 0.85 | 0.85 | 1.25 | 0.81 | 0.82 | 0.96 | 0.84 |
|  | | [0.78 - 2.00] | [0.78 - 2.00] | [0.62 - 1.17] | [0.62 - 1.17] | [0.64 - 1.17] | [0.64 - 1.17] | [0.76 - 1.27] | [1.04 - 1.56] | [0.68 - 1.05] | [0.68 - 1.05] | [0.80 - 1.96] | [0.61 - 1.08] | [0.64 - 1.04] | [0.75 - 1.22] | [0.65 - 1.09] |
| **Caregiver’s marital status** | |  |  |  |  |  |  |  |  |  |  |  |  |  |  |  |
| **(Reference: Married)** | |  |  |  |  |  |  |  |  |  |  |  |  |  |  |  |
| Single | | 0.78 | 0.78 | 0.51 | 0.51 | 0.46* | 0.46* | 0.49* | 0.49* | 0.62 | 0.62 | 0.8 | 0.5 | 0.43* | 0.47* | 0.58 |
|  | | [0.37 - 1.64] | [0.37 - 1.64] | [0.20 - 1.29] | [0.20 - 1.29] | [0.24 - 0.90] | [0.24 - 0.90] | [0.27 - 0.89] | [0.27 - 0.89] | [0.21 - 1.87] | [0.21 - 1.87] | [0.37 - 1.76] | [0.21 - 1.23] | [0.25 - 0.74] | [0.25 - 0.88] | [0.21 - 1.62] |
| **Caregiver’s schooling level** | |  |  |  |  |  |  |  |  |  |  |  |  |  |  |  |
| **(Reference: Less than high school)** | |  |  |  |  |  |  |  |  |  |  |  |  |  |  |  |
| High School & more | | 1.01 | 1.01 | 1.82* | 1.82* | 0.73 | 0.54 | 0.89 | 0.89 | 1.51 | 1.51 | 1.03 | 1.95* | 0.72 | 0.89 | 1.62 |
|  | | [0.65 - 1.57] | [0.65 - 1.57] | [1.32 - 2.51] | [1.32 - 2.51] | [0.37 - 1.43] | [0.28 - 1.05] | [0.59 - 1.33] | [0.59 - 1.33] | [0.97 - 2.34] | [0.97 - 2.34] | [0.67 - 1.58] | [1.20 - 3.18] | [0.38 - 1.36] | [0.64 - 1.25] | [0.94 - 2.77] |
| **Household socioeconomic status** | |  |  |  |  |  |  |  |  |  |  |  |  |  |  |  |
| **(reference: Low)** | |  |  |  |  |  |  |  |  |  |  |  |  |  |  |  |
| Medium | | 0.94 | 0.94 | 0.8 | 0.8 | 2.07* | 4.04* | 0.76 | 0.76 | 1.1 | 1.50* | 0.94 | 0.70* | 2.04* | 0.73* | 1.07 |
|  | | [0.60 - 1.47] | [0.60 - 1.47] | [0.58 - 1.10] | [0.58 - 1.10] | [1.21 - 3.54] | [2.57 - 6.35] | [0.58 - 1.01] | [0.58 - 1.01] | [0.87 - 1.38] | [1.08 - 2.07] | [0.58 - 1.52] | [0.50 - 1.00] | [1.21 - 3.44] | [0.53 - 1.00] | [0.85 - 1.34] |
| High | | 1.35 | 1.35 | 0.95 | 0.95 | 2.54* | 5.62* | 1.3 | 1.3 | 1.08 | 1.77* | 1.27 | 0.82 | 2.40* | 1.22 | 1.04 |
|  | | [0.82 - 2.21] | [0.82 - 2.21] | [0.56 - 1.61] | [0.56 - 1.61] | [1.29 - 5.00] | [3.70 - 8.52] | [0.86 - 1.96] | [0.86 - 1.96] | [0.75 - 1.55] | [1.21 - 2.59] | [0.74 - 2.17] | [0.44 - 1.53] | [1.20 - 4.80] | [0.79 - 1.88] | [0.69 - 1.56] |
| **Household location: Urban/rural** | |  |  |  |  |  |  |  |  |  |  |  |  |  |  |  |
| **(reference: Rural)** | |  |  |  |  |  |  |  |  |  |  |  |  |  |  |  |
| Urban | | 1.57* | 1.57* | 1.22* | 1.22* | 1.1 | 0.66* | 1.27* | 1.27* | 1.40* | 1.16 | 1.58* | 1.29* | 1.14 | 1.34* | 1.42* |
|  | | [1.21 - 2.04] | [1.21 - 2.04] | [1.00 - 1.48] | [1.00 - 1.48] | [0.86 - 1.40] | [0.47 - 0.93] | [1.08 - 1.50] | [1.08 - 1.50] | [1.15 - 1.69] | [0.96 - 1.41] | [1.20 - 2.08] | [1.08 - 1.53] | [0.91 - 1.43] | [1.15 - 1.56] | [1.20 - 1.68] |
| **Age group** | |  |  |  |  |  |  |  |  |  |  |  |  |  |  |  |
| **(reference: 2-7 years old)** | |  |  |  |  |  |  |  |  |  |  |  |  |  |  |  |
| 8-12 years old | | 3.33* | 3.33* | 3.17* | 3.17* |  |  |  |  |  |  | 3.59* | 3.16* |  |  |  |
|  |  | [2.67 - 4.16] | [2.67 - 4.16] | [2.62 - 3.84] | [2.62 - 3.84] |  |  |  |  |  |  | [2.70 - 4.77] | [2.56 - 3.89] |  |  |  |
| 13-17 years old | | 3.06* | 3.06* | 2.27* | 2.27* |  |  |  |  |  |  | 3.17* | 2.39* |  |  |  |
|  |  | [2.25 - 4.16] | [2.25 - 4.16] | [1.38 - 3.74] | [1.38 - 3.74] |  |  |  |  |  |  | [2.25 - 4.45] | [1.52 - 3.76] |  |  |  |
| **Sex** | |  |  |  |  |  |  |  |  |  |  |  |  |  |  |  |
| **Reference: female** | |  |  |  |  |  |  |  |  |  |  |  |  |  |  |  |
| Male | |  |  |  |  | 1.65* | 1.65* | 1.19 | 1.80* | 1.02 | 1.02 |  |  | 1.66* | 1.2 | 1.07 |
|  |  |  |  |  |  | [1.33 - 2.05] | [1.33 - 2.05] | [0.87 - 1.62] | [1.40 - 2.32] | [0.65 - 1.60] | [0.65 - 1.60] |  |  | [1.32 - 2.09] | [0.86 - 1.67] | [0.67 - 1.71] |
| **Child’s physical activity** | |  |  |  |  |  |  |  |  |  |  |  |  |  |  |  |
| **(hours per week)** | | 0.99* | 0.99* | 0.98* | 0.98* | 0.96* | 0.96* | 0.98* | 0.98* | 0.99* | 0.99* | 0.98* | 0.98* | 0.96* | 0.98* | 0.99* |
|  | | [0.98 - 0.99] | [0.98 - 0.99] | [0.98 - 0.99] | [0.98 - 0.99] | [0.94 - 0.99] | [0.94 - 0.99] | [0.97 - 0.99] | [0.97 - 0.99] | [0.98 - 1.00] | [0.98 - 1.00] | [0.97 - 1.00] | [0.98 - 0.99] | [0.93 - 0.99] | [0.96 - 0.99] | [0.98 - 1.00] |
| **Child’s Screen time** | |  |  |  |  |  |  |  |  |  |  |  |  |  |  |  |
| **(hours per week)** | | 1.02* | 1.02* | 1.01 | 1.01 | 1.03* | 1.03* | 1.03* | 1.03* | 1 | 1 | 1.02* | 1.01 | 1.04* | 1.03* | 1 |
|  | | [1.00 - 1.04] | [1.00 - 1.04] | [0.99 - 1.03] | [0.99 - 1.03] | [1.03 - 1.04] | [1.03 - 1.04] | [1.02 - 1.04] | [1.02 - 1.04] | [0.99 - 1.02] | [0.99 - 1.02] | [1.00 - 1.04] | [0.99 - 1.03] | [1.03 - 1.04] | [1.02 - 1.04] | [0.99 - 1.02] |
| **Constant** | | 0.05* | 0.02* | 0.21* | 0.08* | 0.10* | 0.01* | 0.05* | 0.01* | 0.08* | 0.02* | 0.05* | 0.23* | 0.11* | 0.05* | 0.07* |
|  | | [0.03 - 0.08] | [0.01 - 0.03] | [0.15 - 0.30] | [0.05 - 0.13] | [0.05 - 0.19] | [0.00 - 0.01] | [0.03 - 0.09] | [0.00 - 0.02] | [0.06 - 0.11] | [0.02 - 0.03] | [0.03 - 0.08] | [0.15 - 0.35] | [0.06 - 0.23] | [0.02 - 0.10] | [0.05 - 0.11] |
| **Observations** | | 4476 | | 4626 | | 3496 | | 2,699 | | 2907 | | 4476 | 4626 | 3496 | 2,699 | 2907 |
|  |  | [6,486,408] | | [6,912,935] | | [4,844,922] | | [4,350,232] | | [4,022,960] | | [6,486,408] | [6,912,935] | [4,844,922] | [4,350,232] | [4,022,960] |

Table A4. Mlogit for children obesity status and caregiver’s patience level, and time-consistency. Using BMI as continuous variable

| **Odds Ratio** | |  |  |  |  |
| --- | --- | --- | --- | --- | --- |
| **(Normal weight as reference)** | | Overweight | Obesity | Overweight | Obesity |
| **Caregiver’s patience** | |  |  |  |  |
| **(reference: patient)** | |  |  |  |  |
| Medium patience | | 1.72** | 0.98 | 1.70** | 0.98 |
|  | | [1.18 - 2.50] | [0.56 - 1.73] | [1.18 - 2.46] | [0.56 - 1.73] |
| Impatient | | 1.16 | 1.27* | 1.14 | 1.26* |
|  | | [0.78 - 1.72] | [1.02 - 1.58] | [0.78 - 1.65] | [1.02 - 1.55] |
| **Caregiver’s time consistency** | |  |  |  |  |
| **(Reference: Time consistent)** | | 1.05 | 2.37** | 1.04 | 2.36** |
| Present bias | | [0.69 - 1.59] | [1.51 - 3.73] | [0.70 - 1.55] | [1.48 - 3.78] |
|  | | 1.18 | 1.44* | 1.18 | 1.44+ |
| Future bias | | [0.81 - 1.70] | [1.00 - 2.07] | [0.83 - 1.68] | [0.99 - 2.09] |
|  | |  |  |  |  |
| **Caregiver’s obesity status** | |  |  |  |  |
| **First degree** | | 1.31** | 1.23* | 1.07** | 1.12** |
|  | | [1.14 - 1.51] | [1.04 - 1.44] | [1.05 - 1.09] | [1.09 - 1.15] |
| Second degree | | 1.00** | 1 |  |  |
|  | | [0.99 - 1.00] | [1.00 - 1.00] |  |  |
|  | |  |  |  |  |
| **Caregiver’s age group** | |  |  |  |  |
| **(reference 18-39)** | |  |  |  |  |
| 40 and older | | 0.77 | 0.96 | 0.78 | 0.97 |
|  | | [0.55 - 1.07] | [0.77 - 1.20] | [0.58 - 1.07] | [0.77 - 1.22] |
| **Caregiver’s marital status** |  |  |  |  |  |
| **(Reference: Married)** |  |  |  |  |  |
| Single |  | 0.60+ | 0.46 | 0.60+ | 0.46 |
|  |  | [0.35 - 1.01] | [0.17 - 1.24] | [0.36 - 1.02] | [0.17 - 1.25] |
| **Caregiver’s schooling level** |  |  |  |  |  |
| **(Reference: Less than high school)** |  |  |  |  |  |
| High School & more |  | 1.28 | 1.19** | 1.29 | 1.19** |
|  |  | [0.85 - 1.92] | [1.07 - 1.33] | [0.85 - 1.96] | [1.06 - 1.34] |
| **Household socioeconomic status** |  |  |  |  |  |
| **(reference: Low)** |  |  |  |  |  |
| Medium |  | 0.70** | 0.98 | 0.70** | 0.97 |
|  |  | [0.57 - 0.87] | [0.66 - 1.46] | [0.56 - 0.86] | [0.65 - 1.46] |
| High |  | 0.76 | 1.48 | 0.76 | 1.48 |
|  |  | [0.51 - 1.13] | [0.88 - 2.47] | [0.51 - 1.13] | [0.89 - 2.47] |
| **Household location: Urban/rural** |  |  |  |  |  |
| **(Reference: Rural)** |  |  |  |  |  |
| Urban |  | 1.47** | 1.33** | 1.46** | 1.33** |
|  |  | [1.28 - 1.68] | [1.09 - 1.63] | [1.28 - 1.68] | [1.09 - 1.63] |
| **Child’s age group (reference: 2-7)** |  |  |  |  |  |
| 08-dic |  | 2.91** | 3.28** | 2.93** | 3.29** |
|  |  | [2.22 - 3.81] | [2.30 - 4.67] | [2.22 - 3.86] | [2.31 - 4.69] |
| 13-17 |  | 2.52** | 2.39** | 2.53** | 2.41** |
|  |  | [1.65 - 3.86] | [1.43 - 4.02] | [1.65 - 3.86] | [1.44 - 4.03] |
| **Child’s sex (reference: Female)** |  |  |  |  |  |
| Male |  | 1.04 | 1.62** | 1.02 | 1.61* |
|  |  | [0.85 - 1.26] | [1.14 - 2.30] | [0.84 - 1.24] | [1.13 - 2.29] |
| **Child’s physical activity (hours per week)** |  | 0.99** | 0.98** | 0.99** | 0.98** |
|  |  | [0.98 - 0.99] | [0.98 - 0.99] | [0.98 - 0.99] | [0.98 - 0.99] |
| **Child’s Screen time (hours per week)** |  | 1.02** | 1.02** | 1.02** | 1.02** |
|  |  | [1.01 - 1.03] | [1.01 - 1.03] | [1.01 - 1.02] | [1.01 - 1.03] |
| **Constant** |  | 0.00** | 0.00** | 0.01** | 0.00** |
|  |  | [0.00 - 0.00] | [0.00 - 0.00] | [0.01 - 0.03] | [0.00 - 0.00] |
| **Observations** |  |  |  |  |  |
| *Expanded to* |  |  |  |  |  |
